# Impact of an Ultra-Endurance Marathon on Cardiac Function in Association with Cardiovascular Biomarkers

**DOI:** 10.1186/s40798-024-00737-1

**Published:** 2024-06-08

**Authors:** Achim Leo Burger, Claudia Wegberger, Maximilian Tscharre, Christoph C. Kaufmann, Marie Muthspiel, Edita Pogran, Matthias K. Freynhofer, Alexander Szalay, Kurt Huber, Bernhard Jäger

**Affiliations:** 1 3rd Medical Department with Cardiology and Intensive Care Medicine, Clinic Ottakring, Montleartstrasse 37, 1160 Vienna, Austria; 2 Medical School, Sigmund Freud University, Vienna, Austria

**Keywords:** Ultramarathon, Cardiac function, Echocardiography, Cardiovascular biomarkers

## Abstract

**Background:**

Participation in ultra-endurance races may lead to a transient decline in cardiac function and increased cardiovascular biomarkers. This study aims to assess alterations in biventricular function immediately and five days after the competition in relation to elevation of high-sensitivity cardiac Troponin I (hs-cTnI) and N-terminal-pro-brain-natriuretic-peptide (NT-proBNP).

**Methods and Results:**

Fifteen participants of an ultramarathon (UM) with a running distance of 130 km were included. Transthoracic echocardiography and quantification of biomarkers was performed before, immediately after and five days after the race. A significant reduction in right ventricular fractional area change (FAC) was observed after the race (48.0 ± 4.6% vs. 46.7 ± 3.8%, *p* = 0.011) that persisted five days later (48.0 ± 4.6% vs. 46.3 ± 3.9%, *p* = 0.027). No difference in left ventricular ejection fraction (LVEF) was found (*p* = 0.510). Upon stratification according to biomarkers, participants with NT-proBNP above the median had a significantly reduced LVEF directly (60.8 ± 3.6% vs. 56.9 ± 4.8%, *p* = 0.030) and five days after the race (60.8 ± 3.6% vs. 55.3 ± 4.5%, *p* = 0.007) compared to baseline values. FAC was significantly reduced five days after the race (48.4 ± 5.1 vs. 44.3 ± 3.9, *p* = 0.044). Athletes with hs-cTnI above the median had a significantly reduced FAC directly after the race (48.1 ± 4.6 vs. 46.5 ± 4.4, *p* = 0.038), while no difference in LVEF was observed. No alteration in cardiac function was observed if hs-cTnI or NT-proBNP was below the median.

**Conclusion:**

A slight decline in cardiac function after prolonged strenuous exercise was observed in athletes with an elevation of hs-cTnI and NT-proBNP above the median but not below.

**Supplementary Information:**

The online version contains supplementary material available at 10.1186/s40798-024-00737-1.

## Key Findings

- Participation in an ultra-endurance marathon with a running distance of 130 km is associated with a transient decline in cardiac function in those athletes with markedly increased N-terminal pro-brain natriuretic peptide and high-sensitivity cardiac troponin I after the race.
- This alteration in cardiac function is not found in athletes with only a minor elevation of these biomarkers.

**Supplementary Information:**

The online version contains supplementary material available at 10.1186/s40798-024-00737-1.

## Background

Physical activity with low to moderate intensity is associated with reduced cardiovascular morbidity and overall mortality [1, 2]. By contrast, ultra-endurance races such as ultra-marathons (UM) with a running duration of substantially more than 42 kms (km) are associated with potential adverse effects on cardiac function [3–5]. Previous studies suggest that ultra-endurance competitions might be associated with a transient alteration of right (RV) and left ventricular (LV) function, including an increase in RV size and biventricular systolic dysfunction after the race [3, 4]. In addition, increased biomarkers indicative of myocyte necrosis, cardiac congestion and inflammation are reported in participants of ultra-endurance events [6, 7]. However, elevation of markers of cardiovascular stress, including high-sensitivity cardiac troponin I (hs-cTnI) and N-terminal-pro-brain-natriuretic-peptide (NT-proBNP), is only detected in some participants, potentially identifying individuals with significant changes of cardiac function during and after the race [6, 8, 9]. So far, data on potential alterations of left and right ventricular function in association with an increase of cardiovascular biomarkers after an ultramarathon are limited [9, 10]. Moreover, it is unclear if changes in cardiac function after an UM are transitory and only present directly after the event, or if theses alterations persist during the days thereafter as well. Thus, the hypothesis of the present study is to investigate if potential acute effects of an UM (130 km) on left and right ventricular function are in relation to an increase of hs-cTnI and NT-proBNP after the competition.

## Methods

### Study Design

**Fig. 1:**
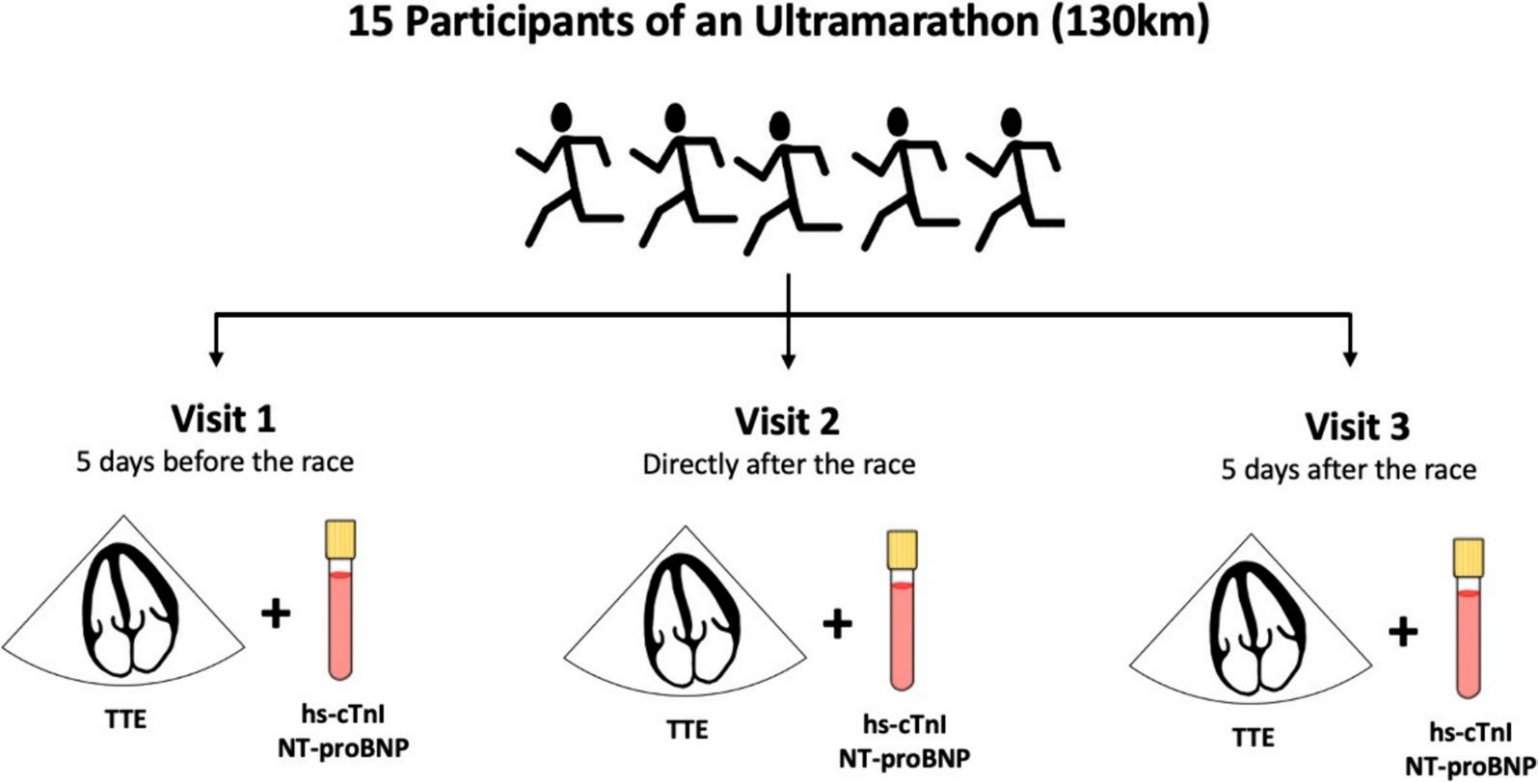
Illustration of study design and conduction (km: kilometers, TTE: transthoracic echocardiography, hs-cTnI: high-sensitivity cardiac Troponin I, NT-proBNP N-terminal-pro brain natriuretic peptide)

The study design has been described previously [6, 7]. This is a prospective, observational study including non-professional athletes. All participants competed in an UM event with a running distance of 130 km. Athletes were invited for three study visits: a baseline visit within five days before the running event; a post-race visit immediately after race, and a follow-up visit five days after the event. Each study visit included a full medical check-up including physical examination, blood sampling, resting 12-lead electrocardiogram, and echocardiography (Fig. 1). The BORG 6–20 scale was used to assess post-race fatigue [11]. Apart from the study visit immediately after the race, all were conducted at the 3rd Medical Department with Cardiology and Intensive Care Medicine, Clinic Ottakring, Vienna.

Individuals were eligible for study inclusion if they were at least 18 years old and had no previous history of cardiac or pulmonary disease (including myocardial infarction, heart failure, valvular disease, congenital heart disease and chronic obstructive pulmonary disease). Potential candidates were required to have previously participated in a regular marathon or ultramarathon. Participants completed the ultra-marathon (“Rundumadum”, Vienna, https://www.wien-rundumadum.at/), which was held in October 2016 and covered 130 km with elevation gains of 1170 m. The track was of asphalt and trail. The time limit given by the organizers to complete the race were 25 h. Written informed consent was obtained from all study participants before any study procedure was performed. The study was conducted in accordance with the Declaration of Helsinki and was approved by the local ethics committee (EK-16-057-VK, 11/05/2016). Wegberger et al. [6] and Kaufmann et al. [7] previously reported data of this study cohort with respect to biomarkers of cardiovascular stress, iron homeostasis and inflammation. Norm values for NT-proBNP are 0–125 ng/L. The upper reference limit (99th percentile) of hs-cTnI is 0.045 ng/mL. The present study resembles a post-hoc analysis investigating the cardiac function by echocardiography of all participants and their association with cardiac biomarkers.

### Study Population

Study recruitment was performed by an open invitation letter published on the official website and regular newsletters of the organizers of the “Rundumadum” Vienna run. Athletes replied voluntarily to the announcement of our study and were invited to the baseline visit if fulfilling all inclusion criteria, being ≥ 18 years, of good health without significant comorbidities and prior participation in a regular marathon or an UM.

### Echocardiographic Exam

A standardized resting echocardiographic exam was performed in left lateral decubitus position at all study visits by experienced operators. Echocardiographic examination was performed using two portable echocardiographs (Vivid iq, General Electrics, WI, USA), equipped with a M4S 1.5–4.0 MHz transducer. Post-exam analyses were performed using a dedicated software (EchoPac, GE, USA). Left atrial volume was assessed using the end-systolic four- and two-chamber views and were indexed to the body surface area (LAVI). Left ventricular ejection fraction (LVEF) was calculated by the Simpson equation using apical four and two-chamber views. Averaged left ventricular global longitudinal strain (GLS) was measured from apical two-, three- and four-chamber views. For right heart assessment a modified apical four-chamber view was acquired. Right ventricular fractional area change (FAC) was measured using end-diastolic and end-systolic areas. Right ventricular global longitudinal free wall strain (GFWS) was assessed using a four-chamber view. All parameters were assessed over three cardiac cycles and then averaged.

### Statistical Analysis

Categorical variables are depicted as counts and percentage. Parametric variables are depicted as mean and standard deviation (SD). The Shapiro–Wilk test was used to check for normality. For comparison of echocardiographic parameters obtained before the UM, immediately and five days after the race, repeated measures analysis of variance (ANOVA) was applied. Fisher’s least significant difference method was used as a post-hoc test for pairwise comparison. The study population was stratified according to the median plasma levels of hs-TnI and NT-proBNP to compare cardiac function in participants with or without substantial biomarker elevation. All statistical analyses were 2-tailed, and a p-value < 0.05 was required for statistical significance. All statistical analyses were performed with SPSS 27.0 (SPSS Inc., Chicago, IL, USA).

## Results

A total of 16 athletes provided informed consent and were recruited for this study. One participant had to be excluded from the final analysis due to non-completion, resulting in a final study population of 15 athletes for full analysis. The mean age of the study population was 42.9 ± 8.0 years with one (6.7%) female participant. All athletes were amateur marathon runners, but had previously completed several UM (n = 11.9 ± 7.8). Participants were in healthy clinical condition without prior cardiovascular or pulmonary comorbidities and no history of smoking. Athletes completed the UM in a mean of 956 min (± 131 min) time with an average speed of 8.0 ± 1.3 km/h, respectively. The level of fatigue quantified with the BORG scale was 15.8 ± 1.6 for the UM (Table 1).

**Table 1:** Baseline Characteristics of the study population (n = 15)

	Mean ± SD or n (%)	Range
Age	42.9 ± 8.0	23–57
Sex (male)	14 (93.3)	
Height, cm	176 ± 8	158–190
Weight, kg	74.2 ± 12.5	50–92
BMI, kg/m^2^	23.8 ± 2.8	20.0–28.2
Total body water, %	40.5 ± 5.3	27.7–47.5
Appendicular muscle mass, kg	8.7 ± 0.6	7.4–9.6
Training per week, km	62.6 ± 30.0	20–120
Running History, years	14.8 ± 9.2	6–34
UM completion time, min	956 (± 131)	726–1221
Average running speed, km/h	8.0 ± 1.3 km/h	6.4–10.7
BORG Scale, points	15.8 ± 1.6	12–18
Hematocrit, %	43.04 ± 2.91	38.2–48.0

BMI body mass index, cm centimetres, dL decilitres, h hours, kg kilograms, km kilometres, min minutes, mg miligramms, m^2^ squaremetres, SD standard deviation, UM ultramarathon

### Changes in Echocardiographic Parameters after the Ultramarathon

The mean LVEF of the study population before the UM was 57.0 ± 6.7%, with a mean GLS of − 19.0 ± 1.8 and a LAVI of 28.4 ± 7.3 ml/m^2^. No significant change in left ventricular function or LAVI was observed after the UM (Table 2). Right ventricular function quantified as FAC was significantly reduced directly after the UM (48.0 ± 4.6 vs. 46.7 ± 3.8, *p* = 0.030), whereas GFWS (− 26.4 ± 2.4 vs. − 24.8 ± 2.7, *p* = 0.766) was similar before and after the UM. The exercise-induced reduction in FAC persisted five days after the UM (48.0 ± 4.6 vs. 46.3 ± 3.9, *p* = 0.027), while GFWS remained unchanged (− 26.4 ± 2.4 vs. − 27.0 ± 2.7, *p* = 0.150).

**Table 2:** Echocardiographic parameters before (Visit 1), directly after the UM (Visit 2) and five days after the UM (Visit 3)

	Visit 1	Visit 2	Visit 3	*p* Value (overall)	*p* Value (V1 vs. V2)	*p* Value (V1 vs. V3)
LVEF	57.0 ± 6.7	55.4 ± 5.7	55.2 ± 4.8	0.510	0.242	0.324
GLS	− 19.0 ± 1.8	− 19.2 ± 1.9	− 18.9 ± 2.0	0.975	0.897	0.926
LAVI	28.4 ± 7.3	28.7 ± 7.6	30.8 ± 6.9	0.639	0.480	0.457
FAC	48.0 ± 4.6	46.7 ± 3.8	46.3 ± 3.9	0.027	0.030	0.027
GFWS	− 26.4 ± 2.4	− 24.8 ± 2.7	− 27.0 ± 2.7	0.263	0.766	0.150

FAC fractional area changes, GFWS global free wall strain, GLS global longitudinal strain, LAVI left atrial volume index, LVEF left ventricular ejection fraction, V visit;

### Echocardiographic Parameters in Relation to Cardiovascular Biomarkers

A significant increase in hs-cTnI and NT-proBNP was observed directly after the UM, which normalised within the following five-day period as previously shown [6]. Participants were stratified according to the median biomarker concentration of hs-cTnI (0.056 ng/ml [IQR: 0.022–0.104] and NT-proBNP (723 ng/L [IQR: 378–1152] assessed immediately after the UM (Table 3 and 4). Participants with plasma levels above the median NT-proBNP had a significantly reduced LVEF (60.8 ± 3.6% vs. 56.9 ± 4.8%, *p* = 0.030) directly after the UM that persisted until day five (60.8 ± 3.6% vs. 55.3 ± 4.5%, *p* = 0.007). Right ventricular function in terms of FAC was not significantly altered in the overall comparison of the three measurements (*p* = 0.184, Table 4), but a significant reduction in FAC was observed five days after the race compared to the baseline value in patients with NT-proBNP above the median (48.4 ± 5.1% vs. 44.3 ± 3.9%, *p* = 0.044). No significant changes in GLS, GFWS or LAVI were observed in relation to NT-proBNP (Tables 3 and 4).

**Table 3:** Echocardiographic parameters in relation to plasma levels below the median of (a) hs-cTnI and (b) NT-proBNP

	Visit 1	Visit 2	Visit 3	*p* Value (overall)	*p* Value (V1 vs. V2)	*p* value (V1 vs. V3)
(a) < median hs-cTnI						
LVEF	56.0 ± 6.2	53.1 ± 6.6	53.5 ± 5.1	0.202	0.115	0.128
GLS	− 19.7 ± 2.0	− 19.4 ± 1.5	− 19.1 ± 1.5	0.678	0.610	0.786
LAVI	24.9 ± 4.0	22.7 ± 2.5	27.1 ± 5.2	0.558	0.233	0.505
FAC	47.5 ± 5.6	45.7 ± 2.4	46.7 ± 6.1	0.192	> 0.05	> 0.05
GFWS	− 27.3 ± 1.4	− 25.0 ± 2.4	− 25.2 ± 0.0	0.530	0.530	0.617
(b) < median NT-proBNP						
LVEF	52.7 ± 7.0	53.6 ± 6.5	55.05 ± 5.3	0.530	0.681	0.285
GLS	− 18.4 ± 2.3	− 18.2 ± 1.9	− 19.8 ± 2.1	0.238	0.800	0.279
LAVI	29.3 ± 4.2	27.6 ± 5.7	32.5 ± 4.2	0.520	0.215	0.478
FAC	46.9 ± 4.0	47.0 ± 3.6	48.3 ± 3.1	0.357	0.357	0.477
GFWS	− 24.4 ± 1.7	− 23.5 ± 2.9	− 25.2 ± 1.5	0.718	0.718	0.205

FAC fractional area changes, GFWS global free wall strain, GLS global longitudinal strain, hs-cTnI high sensitivity cardiac Troponin I, LAVI left atrial volume index, LVEF left ventricular ejection fraction, NT-proBNP N-termianl pro-brain natriuretic peptide, V visit;

**Table 4:** Echocardiographic parameters in relation to plasma levels above the median of (a) hs-cTnI and (b) NT-proBNP

	Visit 1	Visit 2	Visit 3	*p* Value (overall)	*p* Value (V1 vs. V2)	*p* Value (V1 vs. V3)
(a) > median hs-cTnI						
LVEF	57.9 ± 7.4	57.4 ± 4.2	56.5 ± 4.4	0.998	0.948	0.946
GLS	− 19.2 ± 1.7	− 19.0 ± 2.3	− 18.7 ± 2.4	0.948	0.835	0.887
LAVI	32.5 ± 8.4	32.4 ± 7.4	33.1 ± 7.1	0.757	0.418	0.768
FAC	48.1 ± 4.6	46.5 ± 4.4	46.1 ± 2.8	0.108	0.038	0.146
GFWS	− 26.0 ± 2.8	− 25.0 ± 3.2	− 27.3 ± 2.8	0.118	0.419	0.079
(b) > median NT-proBNP						
LVEF	60.8 ± 3.6	56.9 ± 4.8	55.3 ± 4.5	0.029	0.030	0.007
GLS	− 19.5 ± 0.9	− 20.1 ± 1.4	− 18.0 ± 1.5	0.109	0.640	0.104
LAVI	27.7 ± 9,5	29.9 ± 9.9	29.3 ± 8.7	0.584	0.409	0.783
FAC	48.4 ± 5.1	46.5 ± 4.3	44.3 ± 3.9	0.184	0.116	0.044
GFWS	− 27 ± 2.1	− 26.2 ± 2.0	− 28.4 ± 2.6	0.720	0.913	0.381

**Fig. 2:**
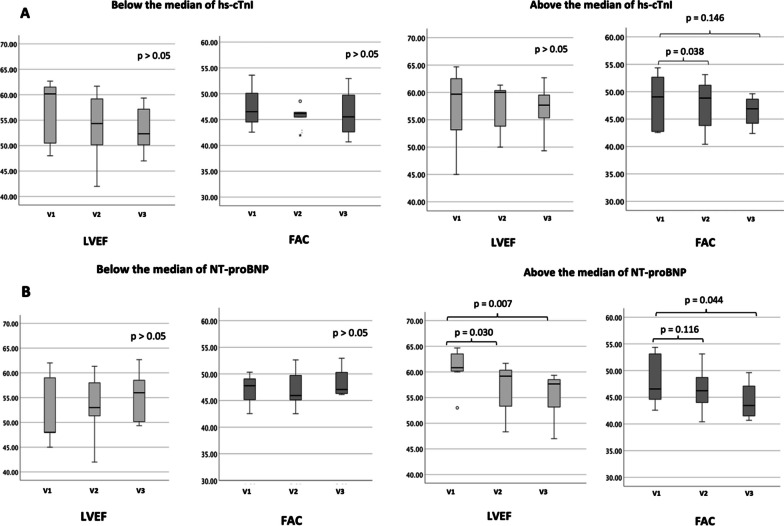
Echocardiographic parameters in relation to plasma levels above or below the median of hs-cTnI (**A**) and NT-proBNP (**B**) before the UM V (Visit) 1, directly after the UM (V2) and 5 days after the UM (V3)

FAC fractional area changes, GFWS global free wall strain, GLS global longitudinal strain, hs-cTnI high sensitivity cardiac Troponin I, LAVI left atrial volume index, LVEF left ventricular ejection fraction, NT-proBNP N-termianl pro-brain natriuretic peptide, V visit;

Participants with plasma levels of hs-cTnI above the median had a significantly reduced FAC directly after the race compared to the baseline visit (48.4 ± 5.1% vs. 46.5 ± 4.3%, *p* = 0.038), which was not observed in the overall comparison of the three measurements (*p* = 0.108). No significant difference in left ventricular function, GFWS or LAVI was observed in relation to plasma levels of hs-cTnI (Tables 3 and 4, Fig. 2).

Stratified to the median finish time of 999 min, participants with a finish time below the median had no different levels of NT-proBNP (812 ± 588 vs. 815 ± 523, *p* = 0.992) or hs-cTnI (0.123 ± 0.125 ng/ml vs. 0.045 ± 0.024, *p* = 0.128) compared to athletes with a finish time above the median.

## Discussion

The main finding of the present study was that only participants of an UM with a biomarker elevation above the median of hs-cTnI and NT-proBNP experienced a decline in cardiac function after the race which was not observed in athletes with biomarker levels below the median. These findings indicate a biomarker-associated impact of prolonged strenuous exercise on cardiac function, which may only be present in participants with a substantial elevation of hs-cTnI or NT-proBNP after the race.

Previous studies indicated that participation in endurance competitions with high cardiac workload over a substantial period may be associated with a transient decline in cardiac function immediately after the race [3–5]. A slight decrease in LVEF and a significant impact on right ventricular function with a reduction of FAC and right ventricular strain was reported in participants of ultra-endurance races, although these findings were not confirmed in other studies [4, 5, 12, 13]. More recently, a study by Cavigli et al. reported no significant changes in biventricular function in participants of an UM of 50km [12]. However, data on potential alterations of right or left ventricular function in participants of an ultra-endurance race in association with an elevation of biomarkers of cardiovascular stress are limited [10, 13].

It is well established that prolonged strenuous exercise may lead to an elevation of cardiovascular biomarkers such as hs-cTnI and NT-proBNP [6, 14–16]. Zebrowska et al. demonstrated in participants of a 24-h ultramarathon that cTnI and NT-proBNP were significantly elevated directly and one day after the race [17]. Systolic left or right ventricular function was not different comparing pre- to post-race evaluation in this analysis, although a significant change in diastolic function was observed [17]. Christensen et al. reported significantly elevated plasma levels of hs-cTnI in participants of a 63-km ultramarathon which was inversely associated with a significant decrease in post-race LVEF [10]. While a significant increase in hs-cTnI and NT-proBNP was seen in the present study [6], reduced cardiac function was not observed in participants with plasma levels of hs-cTnI or NT-proBNP below the median. On the contrary, a decline in right and left ventricular function was only seen in participants with concentrations of NT-proBNP or hs-cTnI above the median as assessed directly after the UM. This finding may indicate that a significant elevation of hs-cTnI and NT-proBNP directly after the race is associated with exercise-induced impairment of cardiac function. Prolonged strenuous exercise may only impact cardiac function in participants with a substantial elevation of hs-cTnI or NT-proBNP after the race.

Increased levels of hs-cTnI can be observed in various clinical conditions leading to cardiac injury, ranging from type 1 myocardial infarction, to tachyarrhythmias or hypertensive emergencies [18]. Prolonged strenuous exercise may lead to elevated hs-cTnI via a similar pathway of cardiac injury [18]. However, Tahir et al. demonstrated that elevation of cTnI after completion of a triathlon was not associated with detectable myocardial oedema by cardiac magnetic resonance imaging [19], indicating that post-exercise elevation of hs-cTnI may be secondary to benign leakage in contrast to myocyte injury or necrosis [19]. In the present study, patients with NT-proBNP levels above the median had a significant reduction of LVEF and FAC after the UM. Potential mechanisms of the transient alterations in cardiac function are not yet fully clarified [20], but may be related to a relative volume overload of the right and left ventricle during prolonged endurance exercise. Increased plasma levels of NT-proBNP may reflect an activation of the cardiac neuroendocrine system secondary to continuous hemodynamic overload [21]. This may explain why the alteration in LVEF and FAC in the present study was more pronounced in relation to NT-proBNP as compared to hs-cTnI. Additional factors may include an increase of markers of systemic stress and a downregulation of cardiac beta-adrenergic receptors after prolonged endurance exercise which might contribute to a transient decline in LVEF and FAC [22–24].

The exercise-induced alteration of LVEF in relation to NT-proBNP in the present study persisted over the early recovery period of five days. Potential long-term effects of these changes in cardiac function are not fully clarified yet, as a few studies reported persistent alterations up to four weeks after prolonged strenuous exercise [25, 26]. Nevertheless, in regards to clinical implications it is important to note that the participants of the present study completed several UM before the conduction of this analysis and retained normal left and right ventricular function. In addition, biomarker elevation normalised within a few days after the event. Thus, a potential impact on long-term cardiac function may be unlikely, although further research is warranted with this respect.

### Limitations

Several important limitations of this study need to be acknowledged. The relative small sample size of the present study and the underrepresentation of female athletes limit the generalizability of the findings and warrant confirmation in larger and more comprehensive study populations. This might be relevant as small effect sizes or potential confounding factors could be outside the reliability of the measures or not big enough to be detected with the power of this study. In addition, this analysis utilized a data-driven cut off (median elevation of biomarkers after the UM) in the lack of established reference values for NT-proBNP and hs-cTnI elevation in this particular setting. Adjustment for multiple comparison was not performed considering the exploratory nature of this study. Some echocardiographic parameters were not available in all athletes at all study visits (Table S3). A requirement for study participation was prior participation in a regular marathon or an UM. However, data on prior participation of an UM is missing in five of the 15 participants (supplementary Table S1). Furthermore, the present study investigated acute effects of ultra-endurance exercise, but no long-term follow-up data is available to draw conclusions on potential long-term effects.

## Conclusions

Participation in ultra-endurance competitions with prolonged strenuous exercise was associated with a slight decline in right and left ventricular function in athletes with an elevation of hs-cTnI or NT-proBNP above the median. This alteration was not observed in participants with hs-cTnI or NT-proBNP levels below the median. Potentially, substantial elevation of hs-cTnI and NT-proBNP directly after an UM is associated with exercise-induced impairment of cardiac function.

### Supplementary Information

Additional file1

## Data Availability

The underlying data of this study are available upon reasonable request to the corresponding author.

## Abbreviations

FAC: Fractional area change
GFWS: Right ventricular global longitudinal free wall strain
GLS: Global longitudinal strain
hs-cTnI: High-sensitivity cardiac Troponin I
km: Kilometres
LAVI: Left atrial volume index
LV: Left ventricular
LVEF: Left ventricular ejection fraction
NT-proBNP: N-terminal-pro brain natriuretic peptide
RV: Right ventricular
SD: Standard deviation
UM: Ultramarathon
