# Supplementary material for: Impact of an Ultra-Endurance Marathon on Cardiac Function in Association with Cardiovascular Biomarkers
Source: Sports Med Open. 2024 Jun 8;10:67. doi: 10.1186/s40798-024-00737-1 (PMC11162405; doi:10.1186/s40798-024-00737-1)
Supplement: Supplementary file 1 — Additional file1 [file 40798_2024_737_MOESM1_ESM.pdf]

Supplementary information

Journal: SPORTS MEDICINE OPEN

Title:

Impact of an ultra-endurance marathon on cardiac function in association with cardiovascular biomarkers

Authors and Affiliations:

Achim Leo Burger[1,2 ‡], Claudia Wegberger[1‡], Maximilian Tscharre[1], Christoph C. Kaufmann[1,2], Marie Muthspiel[1], Edita Pogran[1], Matthias K Freynhofer[1], Alexander Szalay[1], Kurt Huber[1,2], Bernhard Jäger[1,2]

‡ these authors contributed equally

1) 3rd Medical Department with Cardiology and Intensive Care Medicine, Clinic Ottakring, Vienna, Austria

2) Sigmund Freud University, Medical School, Vienna, Austria

Table S1 (supplementary): Individual values per participant for demographic, biomarker and race-related variables.

Pat ID	Age	Sex	BMI [kg/m^2]	NT-proBNP [ng/L]	hs-cTnI [ng/ml]	Finish time [min]	Average velocity (km/h)	Perviously completed UM	BORG Scale
1	48	M	27.47	745	0.084	1036	7.5	4	15.5
2	44	M	22.88	1501	0.180	962	8.1	-	16.0
3	42	M	20.49	479	0.399	881	8.9	7	17.0
4	43	M	28.15	347	0.022	1124	6.9	-	12
5	45	M	24.62	503	0.104	999	7.8	20	17.5
6	43	M	21.24	378	0.088	726	10.7	17	17.0
7	23	M	22.53	236	0.050	865	9.0	5	15
8	46	F	20.03	1912	0.135	808	9.6	9	14
9	33	M	22.27	1152	0.056	1023	7.6	2	17.5
10	53	M	27.17	729	0.047	1135	6.9	6	16
11	39	M	22.18	554	0.059	1057	7.4	15	17
12	38	M	22.58	765	0.015	870	8.9	-	14
13	57	M	24.86	1823	0.026	1221	6.4	32	18
14	42	M	22.93	723	0.015	999	7.8	-	15
15	48	M	28.15	355	0.020	1322	5.9	-	16

BMI body mass index, F female, hs-cTnI high-sensitivity cardiac troponin I, km/h kilometres per hour, M male, min minutes, NT-proBNP N-terminal-pro-brain-natriuretic-peptide, UM ultramarathon, - indicates missing values;

Table S2 (supplementary): Demographic values (age and BMI) stratified to the median of hs-cTnI and NT-proBNP.

	< median hs-cTnI	> median hs-cTnI	p-value	< median NT-proBNP	> median NT-proBNP	p-value
Age	42.1 ± 10.9	43.9 ± 2.9	0.678	40.4 ± 8.2	45.1 ± 7.8	0.274
BMI	24.8 ±2.6	22.7 ± 2.6	0.140	23.9 ± 3.2	23.8 ± 2.6	0.929

Table S3 (supplementary): Availability and missing data of echocardiographic parameters on an individual participant level (0=missing, 1=available):

Pat ID	LVEF	GLS	LAVI	FAC	GFWS
1	1	1	1	1	1
2	1	1	1	1	1
3	1	1	1	1	1
4	1	1	1	1	1
5	1	1	1	1	1
6	1	1	1	1	1
7	1	1	1	1	1
8	1	1	1	0	0
9	1	1	1	1	1
10	1	1	1	1	1
11	1	1	1	0	0
12	1	1	1	1	1
13	1	1	1	1	1
14	1	1	1	1	1
15	1	1	1	1	1

FAC fractional area change, GFWS global free wall strain, GLS global longitudinal strain, LAVI left atrial volume index, LVEF left ventricular ejection fraction
